# PCR Amplification and Transcription for Site-Specific Labeling of Large RNA Molecules by a Two-Unnatural-Base-Pair System

**DOI:** 10.1155/2012/230943

**Published:** 2012-06-26

**Authors:** Michiko Kimoto, Rie Yamashige, Shigeyuki Yokoyama, Ichiro Hirao

**Affiliations:** ^1^RIKEN Systems and Structural Biology Center (SSBC), 1-7-22 Suehiro-cho, Tsurumi-ku, Kanagawa, Yokohama, 230-0045, Japan; ^2^TAGCyx Biotechnologies, 1-6-126 Suehiro-cho, Tsurumi-ku, Kanagawa, Yokohama, 230-0045, Japan; ^3^Department of Biophysics and Biochemistry, Graduate School of Science, The University of Tokyo, 7-3-1 Hongo, Bunkyo-ku, Tokyo 113-0033, Japan

## Abstract

For the site-specific labeling and modification of RNA by genetic alphabet expansion, we developed a PCR and transcription system using two hydrophobic unnatural base pairs: 7-(2-thienyl)-imidazo[4,5-*b*]pyridine (**Ds**) and 2-nitro-4-propynylpyrrole (**Px**) as a third pair for PCR amplification and **Ds** and pyrrole-2-carbaldehyde (**Pa**) for the incorporation of functional components as modified **Pa** bases into RNA by T7 transcription. To prepare **Ds**-containing DNA templates with long chains, the **Ds**-**Px** pair was utilized in a fusion PCR method, by which we demonstrated the synthesis of 282-bp DNA templates containing **Ds** at specific positions. Using these **Ds**-containing DNA templates and a biotin-linked **Pa** substrate (Biotin-**Pa**TP) as a modified **Pa** base, 260-mer RNA transcripts containing Biotin-**Pa** at a specific position were generated by T7 RNA polymerase. This two-unnatural-base-pair system, combining the **Ds**-**Px** and **Ds**-**Pa** pairs with modified **Pa** substrates, provides a powerful tool for the site-specific labeling and modification of desired positions in large RNA molecules.

## 1. Introduction

Site-specific labeling and modification of large RNA molecules provide a wide variety of applications in many areas, such as biochemical and biophysical studies, synthetic biology, *in vitro* evolution, generation of functional nucleic acids, and construction of nanomaterials and biosensors. The site-specific incorporation of functional nucleotide analogs into RNA molecules is performed by chemical synthesis, posttranscriptional modification, and enzymatic incorporation of nucleotide analogs as substrates. Among them, chemical synthesis is a commonly employed method. However, it is only capable of synthesizing small RNA molecules. Other site-specific modifications of RNA, by posttranscriptional modification [1, 2] and enzymatic incorporation using cap or triphosphate analogs [3–6], are limited to terminal modifications of RNA. In contrast to these RNA labeling methods, introducing an artificial, extra base pair (unnatural base pair), as a third base pair, to *in vitro* transcription systems allows the site-specific incorporation of an unnatural base linked with functional groups into desired positions of RNA during transcription mediated by the unnatural base pair. Thus, several unnatural base pairs that function in polymerase reactions have rapidly been developed for site-specific labeling of RNA molecules [7–18].

**Figure 1:**
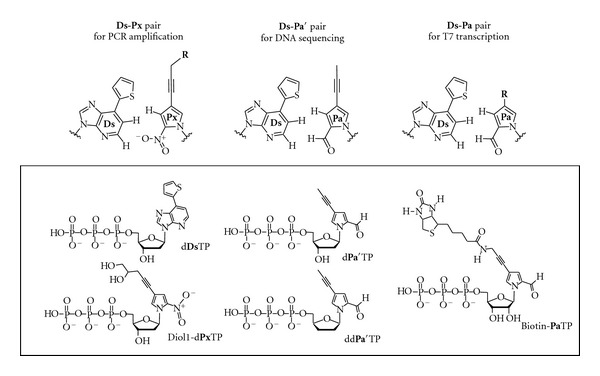
Chemical structures of the **Ds**-**Px**, **Ds**-**Pa′**, and **Ds**-**Pa** pairs. The **Ds**-**Px** pair is employed for PCR amplification to prepare the **Ds**-containing DNA templates. The **Ds**-**Pa′** pair is employed for DNA sequencing, to determine the** Ds** position in the DNA templates. The **Ds**-**Pa** pair is employed for T7 transcription, to incorporate Biotin-**Pa** into RNA opposite **Ds** in the templates. **Px **and** Pa** bases modified with various functional groups (R) can also be used as substrates for PCR and transcription, respectively. The unnatural substrates used in this study are summarized in the box.

**Figure 2:**
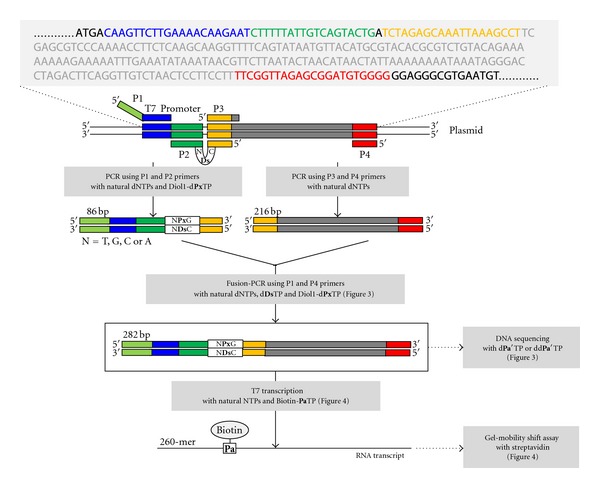
Experimental scheme of PCR amplification and transcription by a two-unnatural-base-pair system for site-specific biotin labeling of RNA molecules. DNA fragments containing the **Ds**-**Px** pair at the internal positions were prepared by a fusion PCR method. The original plasmid sequence, used as the PCR template, is shown at the top. The C**Ds**N sequence (N = T, G, A, or C) in the P2 primer is integrated into the 86-bp DNA fragments. The 282-bp products generated by fusion PCR were used as templates for T7 transcription with Biotin-**Pa**TP.

 Here, for the site-specific incorporation of functional components into large RNA molecules, we report a fusion PCR and transcription system that employs two unnatural base pairs of 7-(2-thienyl)-imidazo [4,5-*b*]pyridine (**Ds**) and 2-nitro-4-propynylpyrrole (**Px**) [19, 20] and **Ds** and pyrrole-2-carbaldehyde (**Pa**) [17] (Figure 1). The **Ds**-**Px** pair exhibits high efficiency and selectivity in PCR amplification as a third base pair. Under optimized conditions, more than 97% of the **Ds**-**Px** pair survives in the 10^28^-fold amplified DNAs through exponential 100-cycle PCR (10 cycles repeated 10 times). In particular, a modified **Px** base, 4-(4,5-dihydroxypent-1-yn-1-yl)-2-nitropyrrole (Diol1**-Px**, Figure 1), has extremely high specificity as a pairing partner of **Ds**, and thus the misincorporation rates of Diol1-d**Px**TP and d**Ds**TP opposite the natural bases in templates during PCR amplification are as low as 0.005% per base pair per replication [20]. Here, we applied the **Ds**-**Px** pair to fusion PCR [21], using small DNA templates containing **Ds** with Diol1-d**Px**TP and d**Ds**TP, to prepare large **Ds-**containing DNA templates for a genetic-expansion transcription system, and generated 282-bp double-stranded DNA fragments containing the **Ds** and Diol1**-Px** pair. Using the **Ds-**containing DNA templates, we performed the site-specific incorporation of Biotin**-Pa**TP, as a functional unnatural base substrate, into 260-mer transcripts by T7 RNA polymerase. In transcription, the **Pa** base is superior to the** Px** base in terms of both the incorporation selectivity opposite **Ds** and the chemical stability of the nucleotide. However, in PCR amplification, the **Ds**-**Px** pair is more selective and efficient than the **Ds**-**Pa** pair, and thus to utilize both pairs' advantages, we developed a two-unnatural-base-pair system, the **Ds**-**Px** pair for fusion PCR, and the **Ds**-**Pa** pair for T7 transcription (Figure 2). By attaching functional groups of interest to the **Pa** base, this fusion PCR and transcription system could be a powerful tool for the site-specific labeling and functionalization of large RNA molecules. 

## 2. Materials and Methods

### 2.1. Materials

 Oligonucleotides containing** Ds** were synthesized with an Applied Biosystems 392 DNA synthesizer, using CE phosphoramidite reagents for the natural and **Ds** bases (Glen Research), and were purified by gel electrophoresis. Oligonucleotides comprising natural bases only were synthesized as described above or purchased from Invitrogen. AccuPrime *Pfx* DNA polymerase, SYBR Gold nucleic acid gel stain, and 10x PBS were purchased from Invitrogen. Streptavidin and silica-membrane columns for PCR product purification (Wizard SV Gel and PCR Clean-Up System) were purchased from Promega. T7 RNA polymerase was purchased from Takara. Toluidine Blue O was purchased from Chroma Gesellschaft Schmidt & Co. The RNA ladder marker (DynaMarker, RNA Low II) was purchased from BioDynamics Laboratory, Inc. The DNA ladder marker (2-Log DNA Ladder) was purchased from New England Biolabs. Unnatural nucleoside triphosphates (d**Ds**TP, Diol1-d**Px**TP, Biotin-**Pa**TP, dd**Pa**′TP, and d**Pa′**TP) were synthesized as described previously [17, 19, 20]. The BigDye Terminator v1.1 Cycle Sequencing Kit was purchased from Applied Biosystems. Centri-Sep spin columns were purchased from Princeton Separations. Natural NTP and dNTP Sets (100 mM solutions: ATP, CTP, GTP, and UTP, and dATP, dCTP, dGTP and dTTP, resp.) were purchased from GE Healthcare. Gel images were analyzed with a bioimaging analyzer, LAS4000 (Fuji Film). The plasmid DNA used for PCR amplification was provided by Dr. Tsutomu Kishi (RIKEN).

### 2.2. Preparation of DNA Templates for T7 Transcription

 To prepare the 282-bp double-stranded DNA fragments containing the **Ds**-**Px** pair as templates for T7 transcription, two steps of PCR using AccuPrime *Pfx *DNA polymerase were performed as follows. First, 86-bp DNA fragments containing the **Ds**-Diol1**-Px** pair were amplified through 25-cycle PCR (50 *μ*L) from 1 ng/*μ*L plasmid DNA template, by using 1 *μ*M 5′-primer (P1: 5′-AAGCT*TAATACGACTCACTATAG* CAAGTTCTTGAAAAC AAGAAT-3′, the T7 promoter region is italicized, and the region complementary to the plasmid is underlined) and 3′-primer (P2: 5′-AGGCTTTAATTTGCTCTAGAC**Ds**NCAGTACTGACAATA AAAAG-3′, N = T, G, A, and C; the region complementary to the plasmid is underlined) and 0.02 U/*μ*L AccuPrime *Pfx* DNA polymerase, in the manufacturer's reaction buffer with 600 *μ*M each natural dNTP, 50 *μ*M Diol1-d**Px**TP, and 2 mM MgSO_4_. The final concentrations of dNTPs and MgSO_4_ were adjusted by adding 300 *μ*M each natural dNTP, 50 *μ*M Diol1-d**Px**TP, and 1 mM MgSO_4_ to the 1xAccuPrime *Pfx* reaction mix, which originally contained 300 *μ*M each natural dNTP and 1 mM MgSO_4_ [20]. PCR conditions were 30 sec at 94°C, 30 sec at 55°C, and 2 min at 65°C per cycle, and the amplified PCR products were purified by gel electrophoresis. As control experiments, we also performed PCR amplification using the P1 and P2 primers without **Ds **(5′-AGGCTTTAATTTGCTCTAGAC**A**TCAGTACTGACAATAA AAAG-3′), in the presence and absence of Diol1-d**Px**TP. A 216-bp double-stranded DNA fragment was amplified by 25-cycle PCR using 1 *μ*M P3 primer (5′-TCTAGAGCAAATTAAAGCCTTCG-3′, the sequence complementary to the P2 primer is underlined) and P4 primer (5′-CCCCACATCCGCTCTAACCGAA-3′) from 1 ng/*μ*L plasmid DNA template in the reaction buffer, containing 600 *μ*M each natural dNTP and 2 mM MgSO_4_, followed by gel purification. Next, we performed fusion PCR (100 *μ*L) with AccuPrime *Pfx* DNA polymerase, using 1 *μ*M each of the P1 and P4 primers, and the purified 86-bp (25 nM) and 216-bp (1 nM) DNA fragments, in the reaction buffer with 600 *μ*M each natural dNTP and 2 mM MgSO_4_, in the presence or absence of 50 *μ*M each of d**Ds**TP and Diol1-d**Px**TP. PCR conditions were 30 sec at 94°C and 3 min at 65°C per cycle. After 25-cycle PCR amplification, the products were purified by passage through silica-membrane columns, according to the manufacturer's instructions. The concentrations of the purified products were determined from their UV absorbance. The purified products were used for DNA sequencing and transcription experiments.

### 2.3. DNA Sequencing

 The cycle sequencing reaction (10 *μ*L) was performed with the Cycle Sequencing mix (4 *μ*L) from the BigDye Terminator v1.1. Cycle Sequencing Kit, 1 *μ*L of 2 *μ*M Sequencing primer (5′-TGACAAGTTCTTGAAAACAAGAAT-3′), 2 *μ*L of 250 *μ*M d**Pa′**TP or dd**Pa′**TP, and 3 *μ*L of 50 nM DNA fragments [20]. The sequencing cycle parameters were 25 cycles of 10 sec at 96°C, 5 sec at 50°C, and 4 min at 60°C. The residual dye terminators were removed with Centri-Sep columns, and the solutions were dried with a centrifugal vacuum evaporator. The residues were resuspended in 3 *μ*L of a formamide/BlueDextran/EDTA loading buffer and analyzed with an ABI 377 DNA sequencer, using a 6% polyacrylamide-6 M urea gel. The sequence peak patterns were analyzed with the Applied Biosystems PRISM sequencing analysis software, v3.2.

### 2.4. Transcription

 Transcription (20 *μ*L) was performed in a buffer containing 40 mM Tris-HCl (pH 8.0), 24 mM MgCl_2_, 5 mM DTT, 2 mM spermidine, and 0.01% Triton X-100, in the presence of 2 mM each NTP, 0 or 2 mM Biotin-**Pa**TP, 100 nM 282-bp DNA template, and 2.5 U/*μ*L T7 RNA polymerase. After an incubation at 37°C for 3 h, the reaction was quenched by adding an equivalent volume of a denaturing solution, containing 10 M urea in 1xTBE. The reaction mixtures were heated at 75°C for 3 min, and 7.5 *μ*L aliquots were fractionated on a 5% denaturing polyacrylamide-7 M urea gel. After electrophoresis, the transcribed products on the gel were stained with toluidine blue and detected by a LAS 4000 imager. For gel-mobility shift assays, the full-length products were purified on a 5% denaturing polyacrylamide-7 M urea gel. 

### 2.5. Gel-Mobility Shift Assay

We detected the biotinylated RNA transcripts (260-mer) by gel-mobility shift assays, using streptavidin. We incubated the mixture (10 *μ*L) of 0.5 pmol transcripts and excess amounts of streptavidin (800 ng) for 1 h at 25°C, in 1x PBS containing 5% glycerol. The biotinylated RNA-streptavidin complexes were separated from the free RNAs on a non-denaturing 8% polyacrylamide gel, and the RNAs on the gel were stained with SYBR Gold and detected by an LAS 4000 imager.

## 3. Results and Discussion

### 3.1. Fusion-PCR Mediated by the Ds-Px Pair for Preparing Long DNA Templates Containing Ds at Desired Positions

To examine fusion PCR [21] using the **Ds**-**Px** pair, we prepared the 282-bp double-stranded DNA (dsDNA) templates containing the **Ds** and Diol1**-Px** pair at internal positions by two PCR steps, using four PCR primers (P1 to P4) and a plasmid DNA, as shown in Figure 2. The P2 primer contains **Ds** and the P3 region. In the first PCR, we used the plasmid as an initial PCR template to introduce the **Ds**-**Px** pair, and prepared two dsDNA fragments (86- and 216-bp) by using AccuPrime *Pfx* DNA polymerase [20]. The 86-bp DNA fragment was amplified by the P1 and P2 primers and corresponds to the 5′ region of the 282-bp template, which contains the T7 promoter sequence in its 5′ region and the **Ds**-**Px** pair in its 3′ region. The 216-bp DNA fragment was amplified by the P3 and P4 primers and corresponds to the 3′ region of the 282-bp template, which comprises natural bases only. Since the P2 and P3 primers share a 20-mer complementary sequence, the 282-bp templates can be prepared by the second PCR step (fusion PCR) using the 86-bp and 216-bp DNA fragments and the P1 and P4 primers.

For preparing the **Ds**-containing 86-bp DNA fragments, we performed 25 cycles of PCR using each of the four P2 primers encoding a 5′-C**Ds**N-3′ sequence (N = T, G, C, or A), in the presence of Diol1-d**Px**TP, as well as the natural dNTPs as substrates and the P1 primer. Each amplified PCR product was purified by gel electrophoresis, to completely remove the original plasmid template. As control experiments, we also performed PCR amplification using a non-**Ds-**containing P2 primer with a 5′-CAT-3′ sequence, in the presence and absence of the unnatural base substrate, Diol1-d**Px**TP. The end-point analysis of the PCR products on the agarose gel revealed no differences among their PCR amplification efficiencies (data not shown). Thus, under the conditions employed here by using AccuPrime *Pfx* DNA polymerase, the nature of the natural base nucleotide at the 5′-neighboring position of **Ds** does not affect the amplification efficiency.

**Figure 3:**
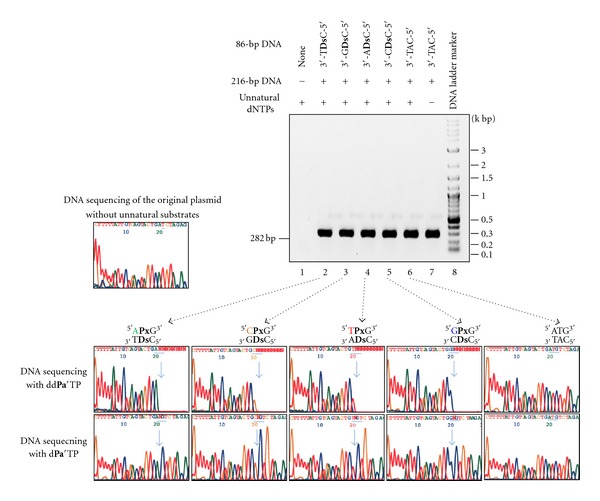
PCR amplification and DNA sequencing of 282-bp DNA templates containing **Ds** at specific positions. After 25-cycle fusion PCR using the P1 and P4 primers and the 86-bp and 216-bp DNA fragments as templates in the presence or absence of d**Ds**TP and Diol1-d**Px**TP, the amplified PCR products were analyzed on a 1% agarose gel. Using the purified PCR products, the sequencing reactions were performed in the presence of dd**Pa′**TP (upper sequencing panels) or d**Pa′**TP (lower sequencing panels). The light blue arrow indicates the unnatural base position. The light blue bar indicates the inserted sequence.

To prepare the full-length 282-bp templates by fusion PCR, we performed 25-cycle PCR using the P1 and P4 primers and the gel-purified 86-bp and 216-bp DNA fragments as templates, in the presence of Diol1-d**Px**TP and d**Ds**TP together with the natural dNTPs as substrates.  Figure 3 shows the agarose gel of the PCR-amplified products. The fusion PCR involving the **Ds**-**Px** pair with all four sequence contexts generated the full-length products with high efficiency, similar to that of the control experiment using the DNA fragments with only the natural base sequence context.

The site-specific incorporation of the **Ds**-**Px** pair into each of the 282-bp DNA fragments was confirmed by dideoxy dye terminator sequencing of the **Ds**-containing DNA strands in the presence of the dideoxyribonucleoside or deoxyribonucleoside triphosphate of 4-propynylpyrrole-2-carbaldehyde (d**Pa′**TP or dd**Pa′**TP, Figure 1), another pairing partner of **Ds **(Figure 3), according to our previously reported method [19, 20]. In the presence of dd**Pa′**TP, the sequencing reaction terminated at the unnatural base position because of the incorporation of dd**Pa′**TP opposite **Ds** in the DNA templates, and thus the following base peaks disappeared. In the sequencing in the presence of d**Pa′**TP, the d**Pa′**TP was incorporated opposite **Ds** in the DNA templates, and the following base peaks appeared, but there is no peak at the unnatural base position because no dye terminator corresponding to the unnatural base was present in the sequencing reaction. By comparing the sequence patterns of the DNA fragment containing 5′-A**Px**G-3′ with that containing 5′-ATG-3′, the differences are clearly observed. These results indicated that the **Ds**-**Px** pair functions well in fusion PCR using AccuPrime *Pfx* DNA polymerase, and from the sequencing analysis, the retention rate of the unnatural base pair in the amplified DNA fragments was more than 97%.

### 3.2. T7 Transcription, Mediated by the Ds-Pa Pair for the Site-Specific Biotinylation of Large RNA Molecules Using a Biotin-Pa Substrate

**Figure 4:**
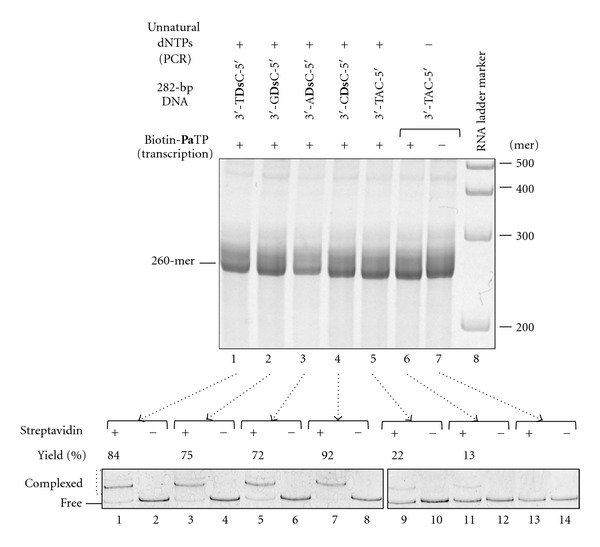
T7 transcription for site-specific biotin labeling of 260-mer transcripts. Transcripts from each 282-bp DNA template containing 3′-T**Ds**C-5′, 3′-G**Ds**C-5′, 3′-A**Ds**C-5′, or 3′-C**Ds**C-5′, in the presence of the natural NTPs (2 mM) and Biotin-**Pa**TP (2 mM), were analyzed on a 5% denaturing polyacrylamide gel (upper gel image). After the purification of each 260-mer transcript, gel-mobility shift assays were performed using streptavidin. Biotinylated RNA-streptavidin complexes were separated from free RNAs on an 8% nondenaturing polyacrylamide gel, and the amounts of the complexes (yields) were determined from the band intensities. The experiments were independently repeated twice, and representative data are shown. The gel-mobility shift values were averaged from two data sets.

Next, we examined the transcription by T7 RNA polymerase, using the 282-bp DNA templates containing **Ds** and Biotin-**Pa**TP as a functional component. Transcription was performed in the presence of 2 mM Biotin-**Pa**TP, as well as 2 mM natural NTPs, at 37°C for 3 h, and the full-length transcripts (260-mer) were analyzed by denaturing polyacrylamide gel electrophoresis (Figure 4). As control experiments, the non-**Ds**-containing DNA templates with the 5′-CAT-3′ sequence, which were PCR amplified with or without the unnatural base substrates, were transcribed with or without Biotin-**Pa**TP. All of the transcription reactions produced similar yields of the 260-mer products.

To characterize the selectivity of the Biotin-**Pa** incorporation into RNA fragments by transcription, we performed gel-mobility shift assays of the biotinylated transcripts in the presence of streptavidin (Figure 4). As for the incorporation site, we already confirmed the site specificity of the Biotin-**Pa** incorporation opposite **Ds** in templates with various sequence contexts [17, 18]. From the gel shift assays, we estimated the biotinylation yields of each transcript (92% of the transcript was biotinylated for the 3′-C**Ds**C-5′ template sequence, 84% for 3′-T**Ds**C-5′, 75% for 3′-G**Ds**C-5′, and 72% for 3′-A**Ds**C-5′). Thus, in contrast to fusion PCR involving the **Ds**-**Px** pair, the biotinylation selectivity depended to some extent on the neighboring bases of **Ds**, and the order of the effective sequence contexts of the 282-bp DNA templates was the same as that of the previously determined effective sequence context of the 35-mer synthetic DNA template for a 17-mer RNA transcript with **Pa** [18]. The biotinylation yields resulted from the sum of the 25-cycle PCR and T7 transcription selectivities. However, the sequencing analysis (Figure 3) indicated that the retention of the unnatural-base-pair in the amplified DNA was more than 97% after 25-cycle PCR, and thus the biotinylation yields actually depended on the transcription selectivities of the **Ds**-**Pa** pair when using each DNA template.

We also assessed the misincorporation of Biotin-**Pa**TP opposite the natural bases in the two-unnatural base pair system. The misincorporation rates of Biotin-**Pa**TP into the transcript using the control 3′-TAC-5′ templates, which were prepared by fusion PCR in the presence and absence of d**Ds**TP and Diol1-d**Px**TP, were 22% and 13%, respectively. Thus, the misincorporations correspond to only 0.096% (for 22% total) and 0.054% (for 13% total) per position in the 260-mer transcript, as calculated by the following equation: *y* = (1 − *x*/100)^260^, where *x*% is the Biotin-**Pa** misincorporation rate opposite the natural bases per position and *y* is the ratio of nonbiotinylated transcript. These misincorporation rates were lower than those of the noncognate natural base pairings, as we previously reported that the misincorporation of biotin-linked UTP opposite G, C, and T in the T7 transcription of a non-A-containing template was around 0.16% per base [17]. In addition, the value of 0.054% is quite consistent with the value (0.06%) that we previously determined by T7 transcription of 152-mer transcripts in the presence of 2 mM Biotin-**Pa**TP and 2 mM natural NTPs [17]. This misincorporation rate also corresponds to the sum of the 25-cycle PCR and T7 transcription selectivities. Thus, the difference between 0.096% and 0.054% was caused by the **Ds** misincorporation into the template strand during 25-cycle PCR amplification with d**Ds**TP and Diol1-d**Px**TP. In the 25-cycle fusion PCR, the PCR primers were mostly consumed, and the theoretical amplification cycle was estimated as ten (2^10^ ≈ 1000 = [primer]/[216-bp DNA fragments]). Therefore, the **Ds **misincorporation opposite a natural base per replication was calculated as approximately 0.004% (≈(0.096% − 0.054%)/10). Consequently, the **Ds **misincorporation into the nontemplate strand would occur, and thus the misincorporation rates of Diol1-d**Px**TP and d**Ds**TP opposite the natural bases in templates during PCR amplification would be 0.008%, which is as high as that of the noncognate natural base pairings.

## 4. Conclusion

In this study, we demonstrated the two-unnatural-base-pair system for the site-specific labeling of large RNA molecules by fusion PCR and T7 transcription. In fusion PCR, more than 97% of the **Ds**-**Px** pair was retained in the amplified 282-bp DNA fragments, and the misincorporation rate of the unnatural bases opposite the natural bases was 0.008% per base per replication. We employed the natural base substrates at relatively high concentrations (600 *μ*M each), as compared to the unnatural base substrates (d**Ds**TP and Diol1-d**Px**TP, 50 *μ*M each) in the fusion PCR, to reduce the misincorporation rate. By using this method, the biotin-linked unnatural base was site-specifically incorporated at predetermined positions of RNA transcripts with selectivities ranging from 72% to 92%, depending on the sequence contexts around the unnatural base. In addition, the misincorporation rate of the biotin-unnatural base opposite the natural bases was around 0.05% per natural base. These selectivity and misincorporation rates were obtained using 2 mM unnatural and natural base substrates in T7 transcription. The incorporation selectivity (90−96%) and the misincorporation rate of Biotin-**Pa** depend on the ratio of Biotin-**Pa**TP to the natural base NTPs in transcription [17]. Thus, the incorporation efficiency and misincorporation rates can be adjusted by changing the concentration ratios between the unnatural and natural base substrates. To reduce the misincorporation of the unnatural base substrate opposite the natural bases, with the sacrifice of the incorporation efficiency of the unnatural base substrate at the desired positions, transcription should be performed with 1 mM unnatural base substrate and 2 mM natural base substrates [12, 17]. Since several functional groups can be attached to the **Pa** base, this method could be applied to a wide range of site-specific labeling and functionalization of large RNA molecules.
